# The epidemiology and factors associated with nocturnal enuresis among boarding and daytime school children in southeast of Turkey: a cross sectional study

**DOI:** 10.1186/1471-2458-9-357

**Published:** 2009-09-22

**Authors:** Ali Gunes, Gulsen Gunes, Yasemin Acik, Adem Akilli

**Affiliations:** 1 Urology Department, Inonu University, Medical School, Malatya, Turkey; 2 Public Health Department, Inonu University, Medical School, Malatya, Turkey; 3 Public Health Department, Firat University, Madical School, Elazig, Turkey; 4 Bozova Government Hospital, Bozova, Sanli Urfa, Turkey

## Abstract

**Background:**

Nocturnal enuresis is an important problem among young children living in Turkey. The purpose of this study was to determine the possible differences in the prevalence of enuresis between children in boarding school and daytime school and the association of enuresis with sociodemographic factors.

**Methods:**

This was a cross-sectional survey. A total of 562 self-administered questionnaires were distrubuted to parents from two different types of schools. One of them was a day-time school and the other was a boarding school. To describe enuresis the ICD-10 definition of at least one wet night per month for three consecutive months was used. Chi-square test and a logistic regression model was used to identify significant predictive factors for enuresis.

**Results:**

The overall prevalence of nocturnal enuresis was 14.9%. The prevalence of nocturnal enuresis declined with age. Of the 6 year old children 33.3% still wetted their beds, while the ratio was 2.6% for 15 years-olds. There was no significant difference in prevalence of nocturnal enuresis between boys and girls (14.3% versus 16. 8%). Enuresis was reported as 18.5% among children attending day time school and among those 11.5% attending boarding school (p < 0.05). Prevalence of enuresis was increased in children living in villages, with low income and with positive family history (p < 0.05). After multivariate analysis, history of urinary tract infection (OR = 2.02), age (OR = 1.28), low monthly income (OR = 2.86) and family history of enuresis (OR = 3.64) were factors associated with enuresis. 46.4% of parents and 57.1% of enuretic children were significantly concerned about the impact of enuresis.

**Conclusion:**

Enuresis was more frequent among children attending daytime school when compared to boarding school. Our findings suggest that nocturnal enuresis is a common problem among school children, especially with low income, smaller age, family history of enuresis and history of urinary tract infection. Enuresis is a pediatric public health problem and efforts at all levels should be made such as preventive, etiological and curative.

## Background

Nocturnal enuresis can be defined as the involuntary passage of urine during sleep beyond the age of anticipated nightime bladder control, after 4-6 y of age [1,2]. It is well known that nocturnal enuresis is a common, genetically complex and heterogeneous disorder among children [3].

According to International Children's Continence Society (ICCS),**i**ntermittent incontinence is urine leakage in discrete amounts. It can occur during the day and/or at night, and it is applicable to children who are at least 5 years old. Enuresis means intermittent incontinence while sleeping. In contrast to the previous terminology, the terms (intermittent) nocturnal incontinence and enuresis are now synonymous[4]. Enuresis can be further categorized into primary nocturnal enuresis or secondary nocturnal enuresis. Primary nocturnal enuresis is therefore bedwetting in a child aged 5 years or more who has never been dry for extended periods, while secondary nocturnal enuresis is the onset of wetting after a continuous dry period of more than 6-12 months[5].

The etiology of enuresis is not completely understood. This condition probably has a multifactor etiology. Most studies have consistently found that the risk factors for enuresis are male gender, smaller age, family history and divorced parents [1,3,6-8]. The overall prevalence of nocturnal enuresis, as well as prevalence of nocturnal enuresis in different age groups, is greatly varied in different countries, ranging from 2.3% to 25% [3] Enuresis is frequently diagnosed among schoolchildren and is an important psychosocial problem both for parents and children. [2]. The relationship between enuresis and behavioural problems has been studied for several decades. Results range from enuretic children having no marked emotional, social or behavioural problems, to enuretic children with a 4.3-times increase in psychological difficulties compared with their non-enuretic peers [9-11].

Although enuretic children seem to have accompanying psychological problems, it must be investigated whether these problems are the results of enuresis or aetiological factors. Nocturnal enuresis is multifactorial, few studies have clarified the pathophysiology of Nocturnal enuresis. Several pathophysiological mechanisms have been proposed, including bladder dysfunction, a small functional bladder capacity, abnormal nyctohemeral vasopressin levels, nocturnal polyuria, and abnormal sleep patterns and arousability. [12,13]

Nocturnal enuresis may cause secondary emotional and social problems in children who continue to wet their bed. A number of etiologic factors have been described to explain this phenomenon[14].

Reports of differences among schools in emotional and social climate were related to changes in behavioral and emotional problems[15]. Social and emotional disturbances were found among students in boarding schools in some studies [16,17]. Our hypothesis was defined as enuresis being more commen in boarding schools than in daytime schools"

Regional Boarding Primary Schools (YIBO) are being opened in Turkey in scarcely populated areas for providing primary education services to the age group in villages and sub-village settlements that do not have schools, and for students from poor families as well.

The purpose of this study was to determine the prevalence of nocturnal enuresis and the association of enuresis with sociodemographic factors in 6 to 16 years old children in Bozova, Urfa in Turkey. In addition, we investigated possible differences in the prevalence of enuresis between children in a Regional Boarding Primary school and a regular primary daytime school.

## Methods

We used a cross-sectional study design to determine the prevalence of enuresis in Bozova, Urfa in Turkey and to investigate its relationship to accompanying sociodemographic factors. This study was carried out in Bozova, Urfa which is the largest populated province of the South East Anotolian Region in Turkey.

The permission was obtained from Primary Health Centre of Bozova considering administrative and clinical governance issues related to the regional health care organisation. A written informed consent form was obtained from the parents, stating the study's objectives.

The schools were selected from two different types. One of them was a day-time school and the other one was a boarding school. There is only one Regional Boarding Primary School in Bozova. One daytime primary school was chosen randomly. One class was chosen among same grade classes randomly in each school. Questionnaires were distributed to all students and they were instructed by the school teachers to take them home to their parents. A brief information leaflet was attached to the questionnaire informing the parent of the voluntary nature of study. The students were instructed to help their to parents. Any parent (mother or father) fill the questionnaires. Students in boarding school go their homes on weekend. The teachers collected questionnaires from children after one week. Those not wishing to participate were recorded as "not responding". The questionnaires were returned for 562 (70%) children. The number of children included in the study from these two schools were similar [286 vs. 276 children].

Questionnaires consisted of two sections. The first section was used to document the background data of the child such as age, gender, type of school, monthly income, parental educational level, father's working status, birth order, family size, presence of other people sleeping in the child's room, inhabitation [living in village or county areas], history of urinary tract infection, constipation and parasitic disease according to their statements. Constipation definion was defined as fewer than one bowel movement a day. This was followed by a question on the presence of bedwetting (see Additional file 1).

For nocturnal enuresis the ICD-10 definition of at least one wet night per month for three consecutive months was used [5]. The second section was completed only when wetting was present. Items included in the second section were the frequency of wetting, family history of wetting, previous therapies and indicators of parental attitudes towards their enuretic child, and whether the child was embarrassed by his wetting. The variables of parental concern and child distress ranged from 1 [a great deal] to 4 [not at all].

Statistical analyses were carried out using the Statistical Package for Social Sciences Chi-square test was used to determine the significant predictive factors for nocturnal enuresis. P-values of < 0.05 were considered to be statistically significant. Age variable was tested for normal distribution. A logistic regression model was applied to estimate the odds ratios (OR) of significant predictive factors for enuresis. Age variable was tested for normal distribution. Variables with p values < 0.05 on univariate analysis were included in the regression model by backward elimination

## Results

A total of 562 children aged between 6 and 16 years were investigated [mean 11.21 ± 2.48]. The overall prevalence of nocturnal enuresis was 14.9% and 84 children with nocturnal enuresis were identified. The prevalence of nocturnal enuresis declined with age. [Table 1].

**Table 1:** Prevalence rate of nocturnal enuresis in children

	**Enuretics**		**Nonuretics**	
**Age**	**n**	**%**	**n**	**%**

6-7	16	32.0	34	68.0
8-9	25	23.4	82	76.6
10-11	24	18.9	103	81.1
12-13	16	10.8	132	89.2
14+	3	2.3	127	97.7

Total	84	14.9	478	85.1

There was no significant difference in prevalence of nocturnal enuresis between boys and girls [14.3% versus 16. 8%]. Enuresis was reported as 18.5% among children attending day time school and 11.5% among those attending boarding school [p < 0.05]. Nocturnal enuresis was primary in 60.7% and secondary in 37.2% of the cases. Secondary enuresis in day time school and boarding school were 42.4% and 37.3%, respectively [p > 0.05]. There was no association between enuresis and parent's education, father's working status, presence of other people sleeping in the child's room, birth order of the child [p > 0.05]. Prevalence of enuresis was more in children living in villages, with low income and with positive family history [p < 0.05] [Table 2].

**Table 2:** Social background and some other characteristics in children with and without nocturnal enuresis

	**Enuretics**		**Nonuretics**			
	**n**	**%**	**n**	**%**	**X**^**2**^	**p**
Gender						
Boys	59	14.3	354	85.7	0.535	
Girls	25	16.8	124	83.2		0.272
School type						
Day-time	51	18.5	225	81.5	5.321	0.014
Boarding school	33	11.5	253	88.5		
Father's education						
≤ 5 years	50	13.9	311	86.1	0.954	
> 5 years	34	16.9	167	83.1		0.196
Mother's education						
≤ 5 years	79	15.0	446	85.0	0.064	
> 5 years	5	13.5	32	86.5		0.514
Father's working status						
Yes	63	13.8	393	86.2	2.430	
No	21	19.8	85	80.2		0.082
Monthly income						
High	8	9.3	78	90.7	9.840	
Medium	43	13.0	287	87.0		
Low	33	22.6	113	77.4		0.007
Room sharing						
None	5	10.6	42	89.4	0.749	
2 or more person	79	15.3	436	84.7		0.266
Inhabitation						
Village	54	18.7	235	81.3	6.541	
County	30	11.0	243	89.0		0.013
Family history of enuresis						
Yes	27	42.2	37	57.8	42.150	
No	57	11.4	441	88.6		0.000
Birth order						
1-3^th^	49	16.6	246	83.4	1.506	
4-6^th^	23	13.8	144	86.2		
≥7^th^	12	12.0	88	88.0		0.471
History of Urinary Tract infection						
Yes	25	21.6	91	78.4	5.016	
No	59	13.2	387	86.8		0.021
Constipation						
Yes	15	24.2	47	75.8	4.687	
No	69	13.8	431	86.2		0.029

Total	84	14.9	478	85.1		562

Enuresis was more in children with history of urinary tract infection, with constipation and with history of parasitic disease [p < 0.05]

After multivariate analysis, history of urinary tract infection [OR = 2.02], age [OR = 1.28], low monthly income [OR = 2.86] and family history of enuresis [OR = 3.64] were factors associated with enuresis.

Overall, 32.1% children with nocturnal enuresis of children [27/84] had a positive family history. In most children, episodes of enuresis occured less than 2 nights per week. Parents were asked about the impact of enuresis on their life and the child's life. 46.4% of parents and 57.1% of enuretic children were significantly concerned about the impact of enuresis. Of the enuretic children, only 11.9% (10 children) had visited a physician [Table 3]

**Table 3:** Possible relationship of different factors in children with nocturnal enuresis

	**n**	**%**
Frequency of wetting		
Every night	27	31.0
2-6 nights per week	21	24.1
< 2 nights per week	39	44.8
Family history		
Yes	27	32.1
No	57	67.9
Children who visited a physician	10	11.9
Therapies		
Tablets/Drugs	9	10.7
Behavioral therapy	1	1.1
Parental concern		
1 (a great deal)	39	46.4
2	20	23.8
3	7	8.3
4 (not at all)	18	21.4
Child's concern		
1 (a great deal)	48	57.1
2	15	17.9
3	8	9.5

4 (not at all)	13	15.5

Visiting a physician was not associated with any risk factors such as as gender, age, inhabitation and severity of wetting [Table 4]

**Table 4:** Visiting to a physician and some characteristics in children with nocturnal enuresis

	**Visit to a physician**		**Not visit to a physician**			
	**n**	**%**	**n**	**%**	**X**^**2**^	**p**
Gender						
Boys	5	8.2	56	91.8	2.404	0.146
Girls	5	20.0	20	80.0		
Severity of wetting						
Severe (everynight)	4	14.8	23	85.2	0.389	0.718
Others	6	10.2	53	89.8		
Ages of children						
6-7 years old	4	23.5	13	76.5	2.921	0.104
Others	6	8.7	63	91.3		
Inhabitation						
Village	7	12.7	48	87.3	0.179	1.000

County	3	9.7	28	90.3		

## Discussion

Nocturnal enuresis is common among younger schoolchildren and its frequency decreases with increasing age. In most countries the prevalence of enuresis among 6-11 year olds is reported as 1.4-28%[1,2]. The prevalence rates of enuresis differ across countries, ranging from 4.3% in Chinese children [18] and 52% in Jamaican primary school children [19]

The overall prevalence of enuresis was found to be 12.95% in children aged 5-16 years from France [20] and 15% in children aged 6-11 years from Saudi Arabia [21].

We found the prevalence of enuresis to be 14.9% in a county, southeast of Turkey. In previous studies reported from different Turkish provinces, the prevalence of enuresis was reported as 11.5-17.5% [1,2,22-25].

The prevalence of enuresis showed a decreasing trend with increasing age of children. This trend is also similar to most reports in the literature [14] Spee-Van der Wekke stated that the prevalence of nocturnal enuresis was 15% in the 5-6-year-old group and 1% in the 13-15-year-old group [14]. Lee *et al*. showed that prevalence of enuresis declined with age from 20.4% in 7-year-olds to 5.6% in 12-year-olds [26]. According to our results, of the 6-7 year olds children, 32% had enuresis, while this ratio was 2.3% of 14 years and older. Byrd et al. reported that the prevalence of enuresis was 33% among children 5 year olds, 18% among 8 year olds and 0.7% among 17 year olds in North America [27].

Nocturnal enuresis is more common and prolonged in boys than in girls [28]. According as our findings, gender did not have a significant effect on the prevalence of enuresis. The general principle about gender, enuresis is more common in boys in the early years but equals out in the latter years [29]. Turkish families living in eastern of Turkey generally enroll to school at an older age. It could be possible, because the mean age of children was 11.2 in this study, we did not found gender differences. Some other studies also showed no gender differences [23,24].

Enuresis was more frequent among children attending the day-time school than the boarding school, although by logistic regression analysis no correlation was found. A possible explanation that enuretic children may not want to attend to boarding school. This is an important population of children to study, whether they sleep in open dormitories or would be more prone to teasing and shaming if enuresis occurred. It might be considered that the children attending boarding school could arouse from sleep easy. Nocturnal enuresis has been related to obstructive sleep-disordered breathing in children. In a community sample of children, those with habitual snoring more often had primary nocturnal enuresis than did those without snoring [30]. A problem noted in children with nocturnal enuresis was difficulty in waking during the night [31]. Many parents complain their bed-wetting children are difficult to be fully awakened. In the study of Tai et al., the ratio of deep sleepers between bedwetting and non-bed-wetting children showed a significant difference [31]. A recent epidemiological study by Neveus et al. reported that most of the dry children were relatively easy to arouse from sleep [32]. It is obvious that waking up is still a problem in enuretics and that some questions remain to be answered on this matter.

In our study, there was no relationship between the enuresis prevalence and the educational level of the father and mother. Spee-Van der Wekke found that the educational level of parents was not significantly related to the prevalance of nocturnal enuresis [14]. In Turkey, Gumus *et al*. showed that the low educational level of parents was associated with nocturnal enuresis[2]. Ozden *et al*. also showed that low education level were significantly associated with enuresis [1]. In our study, most of the parents educational level was low. We found that low socio-economic status of the family was associated with nocturnal enuresis. Chiozza *et al*. found that the prevelance of enuresis was higher in families of low socioeconomic class[8]. Low socioeconomic status is also a risk factor for psychopathology[33].

Enuresis was also significantly more common with village inhabitation than with county inhabitation. This might be related to poor sanitation, lower educational level of parents, and smaller monthly income for village families as compared to those for county families. Gumus et al. [2] Chiozza et al. [8] and Gur et al. [23] also showed that lower educational levels of the parents and lower socioeconomic class were both associated with nocturnal enuresis.

Family history of enuresis was found in 32.1% of enuretic children's families in our study. Enuresis history of the child's mother, father, brothers or sisters has frequently been reported as an accompanying finding in the literature. Furthermore, previous studies reported the prevalence of family history in enuretic children as 22-48%. Twin studies also support a genetic basis for enuresis. [1,2,22]

Rona *et al*., in their study of the population of England and Scotland, found that primary nocturnal enuresis was more likely in a child who was not the first born in the family [34]. In this study, however, birth order was not a significant determinant of nocturnal enuresis. Kanaheswari also showed that birth order was not associated with enuresis [29]

When the logistic regression analysis was applied to risk factors for the bed-wetting in the present study, a significant positive correlation was revealed for low age, low income, history of urinary tract infection and family history of enuresis.

We found association between enuresis and history of urinary tract infections [UTI] and constipation in this study. Kajiwara et al. also found that children with a history of cystitis had a significantly higher rate of nocturnal enuresis than children without such a history [35]. Ozden et al found that recurrent UTI were significantly higher in enuretics when compared to non-enuretics [1]. The reason for this is not clear. However, it has been suggested that the strong contraction of the proximal urethra and pelvic floor muscles might cause UTI by leading to urethrovesical reflux of bacteria in the proximal urethra [36]. Pelvic floor overactivity and bladder dysfunction are thought to simultaneously cause overconstriction of the anal sphincter resulting in constipation [35] Inan et al also found that constipation was more frequent in enuretics [37].

There have been few investigations of the severity of bedwetting in the literature. In our study, 31.0% of children wet everynight. Ozden et al defined 33.3% severe enuresis as bed wetting every night in Turkish children[1]. In southeast Anatolia, the prevalence of "marked" enuresis [at least weekly] was 9.8%. [24] In Karachi, 30% of the children with bed wetting wet every night [38]. Wang et al found that the prevalence of bed-wetting every night was 24.6%[39]. One- third of the children with enuresis wet every night in our study. Our result is consistent with other studies.

In the present study only 11.9% of the children were seen by a physician. This low rate demonstrates that most of the children with enuresis were not treated. Oge et al. from Turkey reported that the families mostly choose the traditional methods in attempt to treat enuresis [25]. In the present study 10.7% of the children were treated with medication provided by physicians. The use of medical treatment is low when compared to other studies. [25,26,29] It may be that among parents few know of the availability of medical treatment.

Parental concern toward the problem of enuresis and the child's concern were studied. Results indicate that 46.4% of parents of nocturnal enuretic children and 57.1% of children consider "it a great deal". Kanaheswari reported that 73% of parents of nocturnal enuresis consider it a problem and 76% of children with nocturnal enuresis embarrassed by their problem [29]. Foxman et al. [40] also found that two-thirds of American parents worried about the symptom, and over half the children were disturbed by the problem. Lee *et al*. reported similar findings in Korean children [26]. In our study, parental concern was a little lower from other studies. It may some relationship to the cultural acceptance of enuresis in southeast of Turkey.

There are some limitations of our study. Questionnaires were filled in children's homes by their parents. This might raise questions about objectivity results is not objectively. Family history of wetting is difficult to estimate. Parents might have given false information their children's bedwetting, bowel habits, arousability. The present study was limited to only one boarding school. It would be desirable to conduct a larger population-based study throughout in more number boarding schools in Turkey.

## Conclusion

In summary, the prevalence rates for enuresis in Bozova, Urfa in Southeast of Turkey were similar to other studies from Turkey and higher than western countries. It may be cultural differences in the achievement of bladder control, and in the attitude of parents to their bedwetting child. Enuresis was more frequent among children attending daytime school when compared to boarding school. Our results with enuresis prevalence and associated factors which were smaller age, low income, family history of enuresis and history of urinary tract infection. We documented that most of the children with enuresis in southeast of Turkey do not have adequate attention about enuresis and most of the enuretic children do not receive professional treatment.

Enuresis is a pediatric public health problem and efforts at all levels should be made such as preventive, etiological and curative. The misconceptions among the parents require health education intervention.

## Competing interests

The authors declare that they have no competing interests.

## Authors' contributions

AA; planned to study, participated in its design and coordination; GG; participated in its design and coordination and writing the article; YA; performed statistical analysis and literature collection, AAkilli; collected data.

All authors read and approved the final manuscript.

## Pre-publication history

The pre-publication history for this paper can be accessed here:



## Supplementary Material

Additional file 1 **Appendix**. Survey Questionnaire. Click here for file
