# Supplementary material for: The epidemiology and factors associated with nocturnal enuresis among boarding and daytime school children in southeast of Turkey: a cross sectional study
Source: BMC Public Health. 2009 Sep 22;9:357. doi: 10.1186/1471-2458-9-357 (PMC2754466; doi:10.1186/1471-2458-9-357)
Supplement: Additional file 1 — Appendix. Survey Questionnaire. [file 1471-2458-9-357-S1.DOC]

###### **Appendix**

**Survey Questionnaire**

**1. part**

1. Is your child’s School □Daytime school □ Boarding school

2. Is your child? □ Male □ Female Child Age……………

3. Education level of mother

□illiterate □ primary incompleted □primary □ secondary □ High school or more

4. Education level of father

□illiterate □ primary incompleted □primary □ secondary □ High school or more

5.Father’s working status □Yes □ No

6. Living people number in your home ………………people

7. Presence of other people sleeping in child’s room □None □ 1 person □ 2 or more

8. Your child’s birth order □ 1 □ 2 □3 □4 □5 □ 6 □ 7+

9. Your inhabitation □ village □ County

10. Monthly income in your home □High □Medium □ Low

11. History of urinary Tract infection of your child □Yes □No

12. History of parasitic disease of your child □ Yes □No

13 . Bowel movements a day □ less than 1 □ 1 □ 2 or more

**2. part**

14.Does your child wets his/her bed during nighttime □Yes □ No

If yes,

15.What is the frequency of bedweeting of your child

□ every night □ if not every night …………times per week

□ if less once per week……….. per month □Less than once per month

16.What is duration of bedwetting consecutive months □ at least 3 months □ less than 3 months

17.Before wetting started, had your child been dry at least 6 months □ Yes □ No

18.Family history of enuresis (mother, father or sibling) □Yes □No

19.Have you visited a physician about your child’s wetting □ Yes □ No

20.If you visited a physician, what type of treatment did your child received?

□Counseling □Tablets/Drugs Restricting drinks □ Waking child at night □Bedwetting alarm bell □ other

21.How much has your child’s wetting concerned you (Please circle the appropriate number)

A great deal 1 2 3 4 Not at all

22.How much distress has wetting caused your child ? (Please circle the appropriate number)

A great deal 1 2 3 4 Not at all
